# The Kumar Technique: A Novel and Effective Approach to Transforaminal Epidural Steroid Injections

**DOI:** 10.7759/cureus.47210

**Published:** 2023-10-17

**Authors:** Caitlin M Gray, Colby Skinner, Terrie Vasilopoulos, Chamara Gunaratne, Jin Choi, Angela Fadil, Sanjeev Kumar

**Affiliations:** 1 Department of Anesthesiology, University of Florida College of Medicine, Gainesville, USA; 2 Department of Anesthesiology, North Florida/South Georgia VA, Gainesville, USA; 3 Department of Orthopaedic Surgery and Sports Medicine, University of Florida College of Medicine, Gainesville, USA; 4 Department of Surgery, University of Florida College of Medicine, Gainesville, USA

**Keywords:** lumbar radiculopathy, fluoroscopy guided, transforaminal epidural steroid injection, lumbar radicular pain, degenerative lumbar spinal stenosis, alternative approach

## Abstract

Background

Transforaminal epidural steroid injections (TFESIs) are widely used as a minimally invasive treatment for lumbar radicular pain. This study presents an alternative approach for lumbar TFESI, the Kumar Technique, which utilizes a more lateral and inferior needle starting point to better align the trajectory of the needle with the neural foramen. We hypothesize the Kumar Technique will result in safer and more effective outcomes than the traditional approach to TFESI. This article was previously presented as a poster at the 2023 University of Florida College of Medicine Celebration of Research on February 27-28, 2023, and as an abstract and poster at the 2023 University of Florida Department of Anesthesiology Celebration of Research on March 29, 2023.

Methods

The charts for 1,424 patients who received lumbar TFESIs were retrospectively reviewed, and patients were stratified into groups receiving either the traditional approach or the Kumar Technique. Outcomes measures included numerical pain scores, measures of functional status and activity limitations, duration of pain relief, and procedural complications.

Results

Compared to the group undergoing the traditional approach, patients receiving the Kumar Technique reported a significantly greater decrease in average pain (-2.3 (95% CI: -3.0 to -1.6) vs -1.1 (95% CI: -1.4 to -0.7)) and maximum pain (-2.4 (95% CI: -3.2 to -1.6) vs -1.3 (95% CI: -1.8 to -0.9)). Patients receiving the Kumar Technique had a significantly greater likelihood of reporting any pain relief (OR: 2.10, 95% CI:1.59 to 2.79) compared to those undergoing the traditional approach. In addition, a greater percentage of patients receiving the Kumar Technique experienced at least one month of pain relief compared to the traditional approach (54% vs 40%; z = 3.85, p < 0.001). The occurrence of complications did not significantly vary between the modified (4.1%) and the traditional (3.0%) approaches.

Conclusions

The Kumar Technique is a modified TFESI approach that allows for improved access to the nerve roots through a more lateral and inferior needle entry point. The analysis supports the benefits of the Kumar Technique with patients experiencing a greater reduction in pain and longer durations of pain relief without increasing the risk of complications.

## Introduction

Low back pain is one of the most commonly reported problems in pain medicine with a lifetime prevalence of 65% to 80% [1-3]. Patients with low back pain experience decreased quality of life and high rates of disability [2]. Consequently, low back pain is the top cause of worldwide productivity loss [2]. The socioeconomic impact of low back pain is exacerbated by the cost of treating it. In the US alone the management of low back pain generates an annual economic burden of over $100 billion [1,2]. Low back pain can have many etiologies, but lumbar radiculopathy is a common age-related complaint with an estimated prevalence of 3% to 5% in the general population [4]. Noninvasive management options can include pharmacologic treatment with nonsteroidal anti-inflammatory drugs and acetaminophen as well as physical therapy [1]. Interventional treatments may be indicated for severe pain refractory to more conservative measures.

Epidural steroid injections are a nonsurgical treatment option for patients with radicular lumbar pain who have failed conservative therapy [4]. The effectiveness of steroid injections relies on the precise delivery of the steroid to the ventral nerve root in the epidural space [5,6]. There are multiple approaches to enter the epidural space under fluoroscopic guidance, including interlaminar, caudal, and transforaminal [3,6]. Transforaminal epidural steroid injections (TFESIs) are the most commonly used option and considered more efficacious than a caudal or interlaminar approach for treating radicular pain, likely due to the TFESI’s needle tip placement resulting in better ventral spread of medication during injection [6-8]. The American Society of Interventional Pain Physicians have given TFESIs Level 1 evidence in the treatment of lumbar radicular pain from disc herniations, and other studies have shown they are effective in treating additional sources of lower back pain, including postherpetic neuralgia [9,10]. However, a recent Cochrane Review indicated moderate quality evidence of only slight efficacy of lumbar ESI citing that many of the studies examined had a risk for bias and further study was needed [11]. These contradictory evaluations spur the need for further evaluation and refinement of the procedure. In addition, TFESIs are also used as a diagnostic tool to determine the specific nerve roots causing lower back or radicular pain [12].

The TFESI procedure involves directing a spinal needle posterolateral to the epidural space at the outer border of the intervertebral foramen, often under fluoroscopic guidance [3,13]. Corticosteroids are then injected into the epidural space immediately adjacent to the exiting nerve root to reduce inflammation and alleviate radicular symptoms. However, studies have shown variability in injection methods among clinicians and there is ongoing debate regarding the safest and most effective approach [7,12]. A previous study has shown that alternative approaches can be more efficacious compared to the traditional approach, with pain reduction having a significant correlation with the ventral spread of medication [14]. However, that study was limited by sample size and did not examine long-term outcomes.

This study introduces a novel lumbar TFESI method with a modified angle of approach. In this modified approach, which we name the “Kumar Technique,” the needle starting point is more lateral and inferior to the traditional approach. Therefore, the needle trajectory better aligns with the neural foramen, improving access into the ventral epidural space. In addition to allowing better access to the ventral epidural space, traditional complications of epidural injections may be minimized by the needle tip staying ventral to all neurovascular structures. Thus, we hypothesize that patients will have greater symptomatic relief and fewer complications compared to the traditional approach.

## Materials and methods

Study design and overview

This study retrospectively evaluated the charts for 1,500 patients who underwent lumbar TFESI procedures at the University of Florida Health Pain Medicine Gainesville between December 2018 and September 2020. The protocol was approved by the University of Florida Institutional Review Board, and individual written informed consent was waived. Patients included in the study were at least 18 years of age who underwent lumbar TFESI. Exclusions included patients whose cases were cancelled or erroneously tagged as having undergone a TFESI during this time. The final number of patient charts analyzed was 1,424.

Patients were deidentified for the purpose of database entry. A retrospective chart review was performed to extract basic demographic information, the lumbar TFESI method, and the identified outcome measures. For data analysis, procedures were stratified into two groups based on the method utilized to perform the lumbar TFESI: those using the traditional approach and those using the Kumar Technique. The procedural method used was determined based on the standard approach of the physician performing the case and confirmed by reviewing the fluoroscopic procedural images.

The Kumar Technique

**Figure 1:**
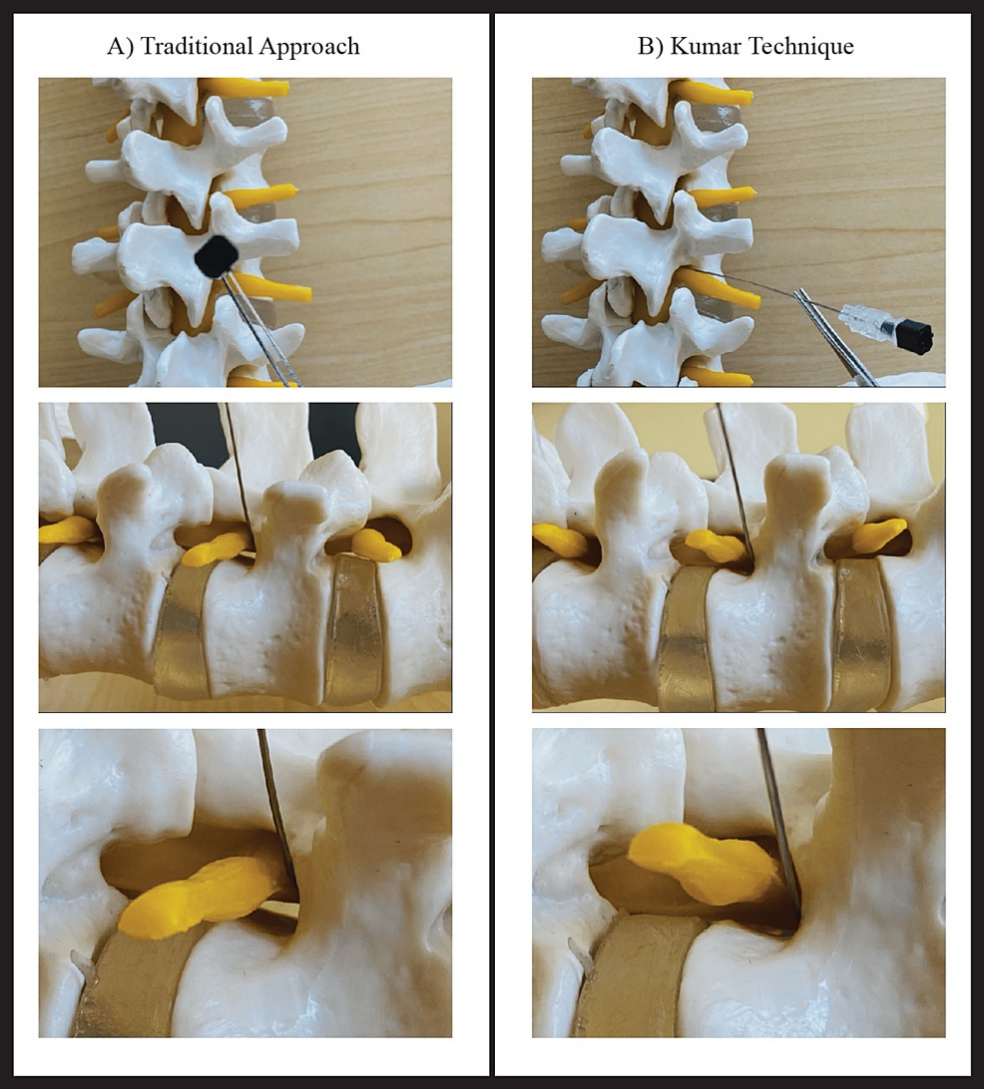
Comparison of the traditional approach and the Kumar Technique

**Figure 2:**
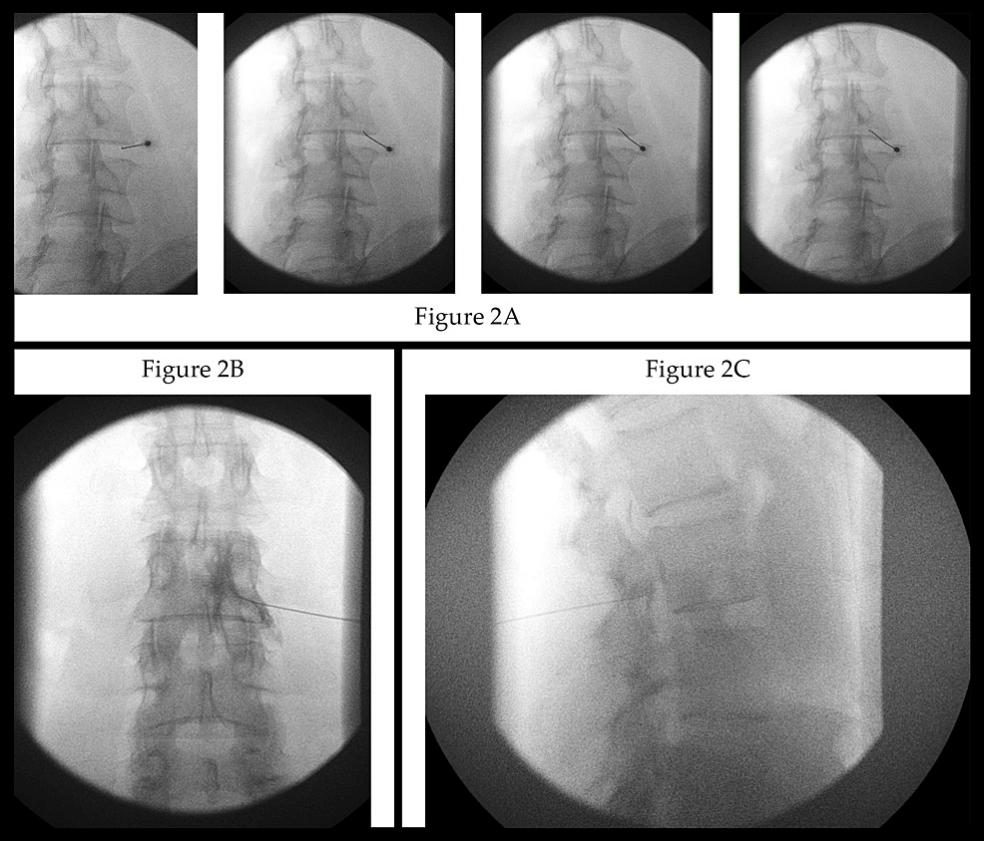
Fluoroscopic Views of the Kumar Technique (A) Trajectory approach: four images showing the needle progression starting with a more lateral and inferior starting point; (B) Anteroposterior view showing needle tip medial to the 6 o’clock position of the pedicle; minimal contrast extravasation; (C) Lateral view showing contrast highlighting the posterior longitudinal ligament, as well as outlining the bulging discs at that level and the level above.

The Kumar Technique starts with a true anteroposterior (AP) fluoroscopic view of the lumbar spine in which the spinous processes of the vertebral bodies are lined up in the midline, equidistant from both pedicles (Figure 1). The C-Arm is then tilted either cephalad or caudad to align the end plates above and below the target disc level, “squaring” them as best as possible. For example, in an L3-L4 transforaminal injection, the C-Arm is tilted until the bottom end plate of the L3 vertebra is square and the top endplate of the L4 vertebra is square and parallel to the bottom end plate of the L3 vertebra. From this position, the C-Arm is rotated 30 degrees towards the side of the injection (right vs left) to produce the classic oblique lumbar spine image (the “Scotty dog” view). A pointer is placed on the skin to mark the bottom edge of the upper vertebral body at the skin level which will become the needle entry point. After adequately anesthetizing the entry point with local anesthetic, a curved tip needle (Havel or Quincke) is advanced through the skin and soft tissues, guided by multiple fluoroscopic images in the oblique view, towards the final radiological target: the junction of the chin and neck of the “Scotty dog.” Periodic AP and lateral C-Arm images may be needed to provide a three-dimensional perspective of the needle advancing in the proper trajectory to its final position, which is the posterior longitudinal ligament (PLL) in the ventral epidural space. The needle tip position is confirmed by contrast injection which outlines the PLL/ventral epidural space with most or all of the contrast inside the spinal canal. There should be no or minimal contrast outside the neural foramen outlining the exiting spinal nerve root. On the AP image, the final needle tip may appear medial to the medial pedicle line, and on the true lateral image, the anterior location of the needle tip should appear in the superior part of the neural foramen at the junction of the pedicle and posterior cortex of vertebral body (Figure 2A-2C).

Outcome measures

To assess subjective pain relief, pain scores measured using a self-assessed numerical rating scale of 0 to 10 were extracted via chart review from the pre-procedure visit, post-procedure at discharge, day-1 follow-up phone call, and the post-procedure follow-up appointment. Measures of functional status included general activity level, walking ability, ability to perform tasks of daily living, enjoyment of life, mood, and sleep. These were extracted from the history of the present illness and review of system sections in the post-procedure follow-up notes. Additionally, PROMIS29 and Oswestry Disability Index (ODI) measurements were documented pre-procedure and post-procedure for each patient. Procedural complications were divided into intraoperative and postoperative complications. Intraoperative complications were extracted directly from the operative report, and these included indications of structural damage during the procedure such as dural punctures and intravascular contrast spread. Post-procedure complications were extracted from postoperative follow-up visit notes and phone calls. These included dural rent, reports of worsening pain or functionality, and new complaints of headaches, weakness, or other complications.

Statistical analysis

Continuous measures were summarized as means with standard deviation, and categorical measures were summarized as counts and percentages. T-tests and chi-square tests were used to assess differences in demographic and clinical characteristics. Group differences in pain were assessed in multiple ways. Linear regression analyses were used to examine group differences in change among both average and maximum pain (before and after the procedure). For these models, group and before-procedure pain were included as independent factors and after-procedure pain was included as the dependent factor. By including before-procedure pain measurements, the model estimates a “residual change score” for pain. A statistically significant effect of group can then be interpreted as an association between group and change in pain. Pain was also assessed by subjective patient reports of pain relief. For this outcome, logistic regression analysis was used, with group as the independent variable and pain relief (yes/no) as the dependent variable. A similar analytic approach was used for post-procedure complications (yes/no). Ordinal logistic regression was used to evaluate group differences in pain relief duration (recoded as a categorical variable: non or worsening, < one week, one week to one month, one to two months, three months+). Regression analyses were also repeated to adjust models for relevant covariates. Effects from linear regression models were quantified as regression efficient (B) with 95% confidence intervals (95% CI). Effects for logistic regression models were quantified as odd ratios (OR) with 95% CIs. P < 0.05 was considered statistically significant. All analyses were performed in JMP Pro 16 (SAS Institute Inc, Cary, USA).

## Results

Overall, n = 1,424 patients were included in the study. Table 1 reports the demographic and clinical characteristics of patients for the entire sample, stratified by approach. Patients undergoing the Kumar Technique were slightly younger (58.5 years ± 15.9) than those receiving the traditional approach (60.8 years ± 15.4, p = 0.012). Patients undergoing the Kumar Technique had higher rates of disc herniation (49.8% vs 43.6%, p = 0.034) and lower rates of stenosis/foraminal narrowing (74.5% vs 79.8%, p = 0.030) compared to those receiving the traditional approach. There were no significant group differences in gender or history of previous back surgeries.

**Table 1:** Demographic and clinical patient characteristics, stratified by approach SD, standard deviation.

Measures	Full Sample (n = 1424)	Kumar Technique (n = 431)	Traditional Approach (n = 993)	P Value
Age, mean years ± SD	60.1 ± 15.6	58.5 ± 15.9	60.8 ± 15.4	0.012
Gender, n (%)				0.571
Female	753/1424 (52.9%)	223/431 (51.7%)	530/993 (53.4%)	
Male	671/1424 (47.1%)	208/431 (48.3%)	436/993 (46.6%)	
Disc herniation, n (%)	633/1392 (45.5%)	210/422 (49.8%)	423/970 (43.6%)	0.034
Stenosis/Foraminal narrowing, n (%)	1088/1392 (78.2%)	316/424 (74.5%)	772/968 (79.8%)	0.030
Previous back surgery, n (%)	322/1384 (23.3%)	100/424 (23.6%)	222/960 (23.1%)	0.852

**Figure 3:**
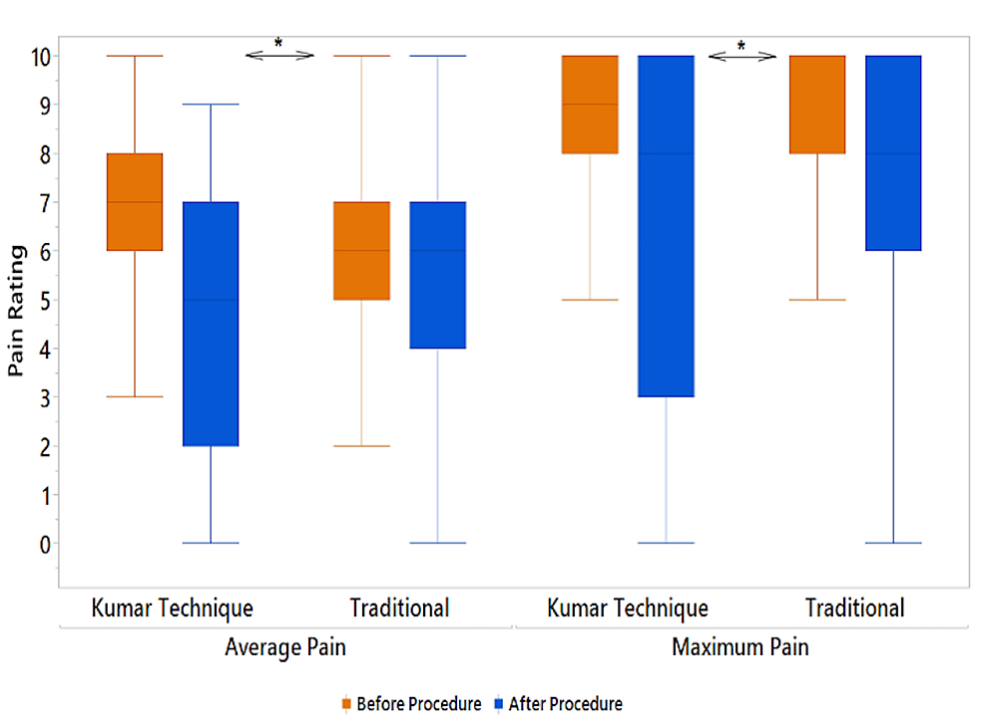
Average and maximum pain before and after procedures for the Kumar Technique and the traditional approach (box plot) * indicates p < 0.05 in change in pain between techniques

**Figure 4:**
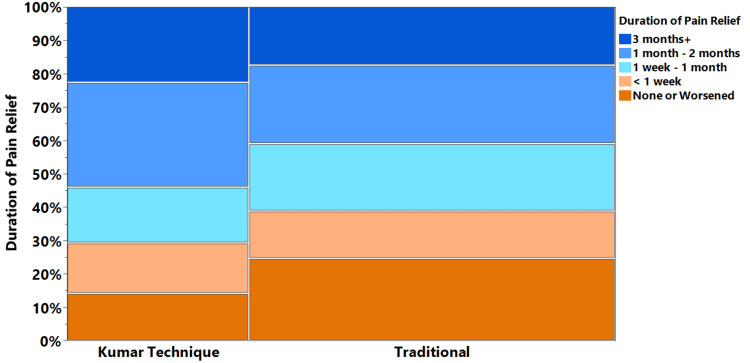
Pain relief durations following procedures for the Kumar Technique and the traditional approach

Table 2 reports results from regression analysis both unadjusted and adjusted for covariates, which include age, disc herniation, and stenosis/foraminal narrowing. The approach emerged as a strong independent predictor of both the degree of pain relief and duration of pain relief. Patients undergoing the Kumar Technique had greater decreases (-2.3, 95% CI: -3.0 to -1.6) in average pain before- and after-procedure compared to those receiving the traditional approach (-1.1, 95% CI: -1.4 to -0.7, Figure 3). Similarly, patients undergoing the Kumar Technique had greater decreases (-2.4, 95% CI: -3.2 to -1.6) in maximum pain before and after the procedure compared to those receiving the traditional approach (-1.3, 95% CI: -1.8 to -0.9, Figure 3). Additionally, patients undergoing the Kumar Technique had 2.2 times greater odds (95% CI: 1.6 to 2.9) of reporting that they had at least some pain relief following the procedure. There was also an association between the procedure and pain relief duration (χ2 = 13.8, p < 0.001). 54% of patients undergoing the Kumar Technique reported pain relief of one month or greater, with 23% reporting pain relief durations of three months or greater (Figure 4). Comparatively, only 40% of patients undergoing the traditional approach reported pain relief of one month or greater (z = 3.85, p < 0.001, Figure 4). Occurrence of complications did not significantly differ between groups (p = 0.277) and was low for both patients receiving the Kumar Technique (4.1%) and the traditional approach (3.0%).

**Table 2:** Effects of the Kumar Technique vs the traditional approach on outcomes for regression models, both unadjusted and adjusted for covariates B, regression coefficient, OR, odds ratio, 95%CI, 95% confidence interval The traditional approach was the reference group for regression analyses. ^a^ Results from linear regression with pain before the procedure included as a covariate to create a “residual change” score. ^b^ Results from logistic regression. ^c^ Adjusted for age, disc herniation, and stenosis/foraminal narrowing.

	Unadjusted			Adjusted		
Outcome	B 95% CI	OR 95% CI	P Value	B 95% CI	OR 95% CI	P Value
Change in average pain^a^	-0.51 -0.87 to -0.15		0.006	-0.51 -0.89 to -0.14		0.008
Change in maximum pain^a^	-0.49 -0.91 to -0.07		0.023	-0.55 -1.0 to -0.11		0.016
Pain relief (yes/no)^b^		2.10 1.59 to 2.79			2.16 1.62 to 2.86	
Complications (yes/no)^c^		1.36 0.72 to 2.54	0.342		1.42 0.76 to 2.66	0.277

## Discussion

A TFESI involves the injection of steroids under fluoroscopic guidance into the epidural space through a neural foramen at vertebral levels that correspond to a patient’s pain distribution [15]. Patients treated with TFESI generally experience low complication rates [16,17]. Moreover, these patients can enjoy a substantial reduction in short- [14,18] and long-term [19,20] low back or radicular pain after TFESI treatment [16,18-21]. However, some patients receive little or no relief in their pain from TFESI [22].

Proper TFESI technique delivers steroids at the site of pathology while minimizing the risk of nerve or vessel damage. The traditional supraneural approach to TFESI utilizes the “safe triangle” for injection, which avoids trauma to the exiting nerve root by placing the needle in the anterosuperior aspect of the intervertebral foramen [8,23]. A rare but potentially catastrophic complication is the injection of the radicular arteries, especially the artery of Adamkiewicz, which can result in spinal cord infarction [8,24]. An alternative method to avoid the radicular arteries is the infraneural approach, also known as Kambin's triangle approach. In this method, the steroid is injected into the posteroinferior aspect of the foramen [25]. However, while this approach reduces the risk of intravascular injection, inadvertent disc injection may occur [26].

The Kumar Technique, when performed properly, should have a lower incidence of these traditional complications because the needle tip remains ventral to all neurovascular structures when approaching the neural foramen. In addition, the needle does not traverse towards the intervertebral disc, minimizing the risk of inadvertent disc penetration. While dural puncture is always a primary concern during epidural procedures, the Kumar Technique curtails this risk as well. The lumbar spine typically has progressively smaller vertebral bodies from L5 to L1, and the dural sac becomes more lateral in the spinal canal closer to the higher levels of the lumbar spine. Despite better alignment of the needle with the neural foramen seeming to increase the risk of dural puncture, the natural decrease in size of the vertebral bodies at higher levels requires an increased needle angle to reach the desired target. In turn, this ensures that the dura is avoided, even when it is more lateral in the spinal canal at the higher levels. Thus, the Kumar Technique is valid for all the lumbar spine levels (L5-S1 through L1-2 levels) and presents a low risk of all major complications resulting from improper needle tip placement.

Under fluoroscopic guidance, contrast injected in the epidural space by the traditional approach quickly dissipates due to the surrounding vasculature. Thus, some of the injectate is “wasted” in the traditional approach as it is not delivered to the site of pathology in the spinal canal and does not remain on the exiting nerve root. Conversely, the trajectory in the Kumar Technique takes the needle safely ventral to the dura and nerve root and lands the tip at the posterior longitudinal ligament (PLL), which is usually an avascular structure except in chronic inflammatory conditions like old disc herniations, where the PLL can get inflamed with neovascularization. The lack of vascularity ensures that the injected steroid remains in place adjacent to the affected nerve root for a longer period, as evidenced by minimal contrast washout on repeat fluoroscopic images. In addition, many lumbar radiculopathies stem from disc bulges/herniations which displace the densely adhered PLL. Delivery of steroids at the PLL ensures precise delivery of medication at the site of pathology with less waste, thereby increasing injection efficacy.

The theoretical benefits of the Kumar Technique are supported by the results of this analysis. Patients treated with the Kumar Technique had significant reductions in immediate post-procedure pain and average pain when compared to patients treated with the traditional TFESI approach. In addition, patients treated with the Kumar Technique experienced pain relief for a longer duration than the traditional approach.

In our retrospective chart review of 1,424 procedures, we sought to determine whether the Kumar Technique was more effective in providing relief from radicular back pain than the traditional approach. We found that patients who received the Kumar Technique had an average immediate post-procedure pain reduction of 2.3 points on a self-reported pain score from 0 to 10. However, patients receiving the traditional approach had an immediate post-procedure pain reduction of just 1.1 points on the same 11-point scale. Results were similar with respect to reduction in average self-reported pain, with patients who received the Kumar Technique experiencing an average reduction of 2.4 points compared to 1.3 points for the traditional approach. Moreover, patients who received the Kumar Technique were 2.2 times more likely to report pain reduction immediately after the procedure.

Another metric of efficacy is the duration of pain relief. The transient nature of pain relief associated with TFESI is well documented and often cited in arguments against the utility of lumbar TFESI. The results of this study found a marked improvement in the duration of pain relief when using the Kumar Technique as compared to the traditional approach. While the majority of patients receiving the traditional approach experienced less than one month of pain relief, 54% of those receiving the Kumar Technique experienced pain relief for greater than one month. Even more notably, 23% of patients receiving the Kumar Technique reported pain relief three months after the procedure.

Our results show that the Kumar Technique is likely a more effective treatment for radicular back pain in most patients than TFESI via the traditional approach. The Kumar Technique allows for improved access to the nerve roots in the epidural space via the neural foramen of the affected vertebral level, thereby decreasing major risks of neurovascular trauma and intravascular injection and minor risks including headache, flushing, and increased back pain [17,27-29]. While the rate of complications did not vary significantly between the Kumar Technique and traditional approach groups, the incidence of complications is generally very low for all TFESI procedures [30]. A larger study may be required to reveal any differences in complication rates, including in types and severity of complications. However, these results suggest that the Kumar Technique provides patients better pain relief without significantly increasing the risk of complications and has become the preferred approach for all Pain Medicine Fellows who train and graduate from our training program including those who have stayed on as faculty at our institution. Although this study did not specifically analyze the relief of radicular symptoms, the Kumar Technique is likely a viable treatment approach for lumbar radiculopathy due to its ability to precisely inject steroids along the PLL.

There are several potential limitations to our study. The average age of patients in this analysis was 58.5 years for the Kumar Technique and 60.5 years for the traditional approach; other TFESI efficacy studies evaluate slightly younger patient populations that average 50 to 55 years old [19,23]. However, previous research has demonstrated age does not affect the extent to which a TFESI will be effective for a patient. Additionally, patients who received the Kumar Technique had slightly higher rates of disc herniation (49.8% vs 43.6%) and slightly lower rates of stenosis/foraminal narrowing (74.5% vs 79.8%) than patients receiving the traditional approach. Patients with disc herniations have previously been shown to have greater reductions in pain from TFESI than those without disc herniation [22]. Similarly, patients who have stenosis/foraminal narrowing typically experience a greater reduction in pain from TFESI than those without [30]. In addition, though our study included patients who had received multiple TFESI procedures, we did not analyze differences in pain relief degree and duration based on whether the procedure was a patient’s first or subsequent procedure, or whether the patient was receiving multiple TFESI injections in one sitting.

In addition, differences in the volume and concentration of steroids injected were not accounted for in our study. Typically, 4 mg of decadron along with 2 cc of normal saline (for a total of 3 cc) is injected at each level for patients having two- to three-level TFESIs performed. However, if a single-level TFESI is performed, 8 mg of decadron is sometimes used; in addition, in severely stenotic patients, sometimes the total volume of injectate is less than 3 cc. However, these differences are the practice standard and should be consistent between the groups performing the traditional approach and the Kumar Technique.

The study is further limited due to the novelty of the technique. Although the Kumar Technique has been mainly used by younger proceduralists at our institution who graduated from our Pain Medicine Fellowship Program, the results may be skewed by the random manner in which patients were chosen to undergo this novel technique versus the traditional technique. Further adoption of the Kumar Technique and future evaluation of the technique across a broader patient population could address this limitation. This emphasizes the importance of the discussion of this new technique as its efficacy can only truly be evaluated when the method is disseminated amongst providers.

Future research could evaluate a younger patient population and control for rates of disc herniation, central stenosis, and foraminal narrowing between each intervention group. In addition, ensuring the same dose of steroids and volume of injectate could be controlled for in future studies. A prospective multicentric randomized control study would be an effective tool for further efficacy and safety evaluations as well as cost analysis of the Kumar Technique.

## Conclusions

TFESI is a widely used treatment for lumbar radicular pain. However, refinements in technique are needed to improve the consistency and efficacy of the procedure. The Kumar Technique is a modified TFESI approach that allows for improved access to the traversing nerve roots and ventral epidural space, through a more lateral and inferior needle entry point. A multicentric, prospective, randomized control study would provide more clear evidence of any objective benefits of this modified approach. This limited current analysis supports the benefits of the Kumar Technique with patients experiencing a greater reduction in pain and longer durations of pain relief without increasing the risk of complications.
